# Supplementary material for: The HINTS examination and STANDING algorithm in acute vestibular syndrome: A systematic review and meta-analysis involving frontline point-of-care emergency physicians
Source: PLoS One. 2022 May 5;17(5):e0266252. doi: 10.1371/journal.pone.0266252 (PMC9070939; doi:10.1371/journal.pone.0266252)
Supplement: Attachment — Submitted filename: Response to Reviewers1.docx [file pone.0266252.s002.docx]

**Comments from Reviewer 1**

- **Comment 1:** *In the discussion section, this reviewer believes that the authors can state the opinion of Vanni, et al who provided their research team of ED physicians with proper training, and found that they perform the STANDING protocol effective.” Not clearly stated, however, is the need for sustained exposure” If they see only one case a week, confidence will remain weak. An increasing number of ED physicians who focus their attention on vertigo/dizziness, thus increasing enthusiasm is noticeable.*

**Response:** Education and training will ensure that emergency physicians can use the STANDING algorithm with the appropriate level of skill and confidence as dizziness or vertigo is a common presentation in emergency settings. Over time, this will help increase collegial interest and enthusiasm. We have amended the text in the Discussion section highlighitn the lack of research in this area (Page 16, Line 287 – 297).

- **Comment 2:** *I also think that the diagnosis may not be straightforward with the first clinical exam. In such cases, Video recording may be the best choice. The cost of video-oculography is not very expensive, and it provides a record of the initial findings, which may become critical in the short term for management. Let us draw an analogy with an Echo obtained in a patient with a heart murmur (the goal is precision). If you agree may want to add.*

*Video- oculography of course would no be needed for BPPV. It is true that local community hospital will need to rely on Telemedicine or refer the patient to a larger facility. It is possible though that common diagnosis such as BPPV or vestibular neuritis could be identify easily by the local ED physician. (If you agree may want to add).*

**Response:** Diagnosis is not always straightforward and VOG-HINTS with telemedicine would be ideal. However, there are multiple barriers which prevent this from being implemented, including cost. Although this topic is beyond the scope of our work, we have amended the text in the Discussion section to discuss this (Page 17, Line 299 - 314).

Whilst diagnostic precision is important in medicine, it is not the immediate goal of a point-of-care ‘screening too’ to facilitate decision making in the emergency setting.

- ***Comment 3:*** *Finally, increase literacy among all future physicians pertaining Neurology and Vestibular Medicine is also predictable in the not so far future. At our School, the curriculum is quite ambitious, and students as they advanced in heir training and early practice will become more aware that this is expected from them. (In the spirit of Education may want to add).*

**Response:** We commend your school’s commitment to developing excellent physicians. However, would like draw attention to the widespread concerns in the literature that physicians, (emergency physicians in particular), are becoming increasingly unskilled and dependent on technology. We have amended the text in the Discussion section to emphasize our concerns (Page 18, Line 315 - 324).

**Comments from Reviewer 2**

- **Comment 1:** *In the Stage 1 Screening the authors state that the excluded irrelevant studies but ignore a lot of papers which use vestibulospinal signs in AVS. “Imbalance” or more properly truncal ataxia deserves a special paragraph, especially if quantify (Lee H, Neurology (2006) 67: 1178-83 – Carmona et al doi: 10.3389/fneur.2016 – Vanni et al doi: 10.3389/fneur.2017)
  a- truncal ataxia is an specific manifestation of the vestibulospinal compromise and its value in the physical examination is the same of the ocular findings
  b- At least in the both side of the spectrum: No ataxia never central, ataxia grade III (unable to walk) is always central (Kiersten L. Gurley, MD1, 2,3 Jonathan A. Edlow, MD, Semin Neurol 2019;39:27–40 - Jonathan A. Edlow, MD, Kiersten L. Gurley, MD,and David E. Newman-Toker* [*-*](https://doi.org/10.1016/j.jemermed.2017.12.024) *-Diagnosing Stroke in Acute Dizziness and Vertigo Pitfalls and Pearls Ali S. Saber Tehrani, MD; Jorge C. Kattah, MD; Kevin A. Kerber, MD, MS; Daniel R. Gold, DO; David S. Zee, MD; Victor C. Urrutia, MD; David E. Newman-Toker, MD, PhD Stroke, March* 2018).

*Other vestibulospinal signs: Bainski asinergia sign, falling from sitting with closed eyes and arm over the chest can help (A New Diagnostic Approach to the Adult Patient with Acute Dizziness.) Edlow JA, Gurley KL, Newman-Toker DE J Emerg Med. 2018 Apr; 54(4):469-483. doi: 10.1016/j.jemermed.2017.12.024. Ideally, have the patient walk unassisted, but for severely nauseated patients too symptomatic to walk, test for truncal ataxia by asking the patient to sit upright in the stretcher with arms crossed. Patients who cannot walk or sit up unassisted are unsafe for discharge and are more likely to have a stroke (or other CNS pathology) rather than vestibular neuritis (27, 44, and 77). Although American emergency physicians are uncomfortable using HINTS testing and instead overuse CT, one study reported that specially trained emergency physicians using these bedside examination elements decreased both CT use and hospitalization.*

**Response:** Thankyou for this observation. We have amended the text to include a section emphasizing the value of other neurological signs, and have linked this to the original HINTS examination and its subsequent variants, in particular, the STANDING algorithm (Page 15, Line 249 – 264).

We believe that our screening criteria were designed and adhered to appropriately to exclude studies which were not relevant to our research question. We were investigating the viability of educating emergency physicians in emergency setting only; we were not reviewing physicians belonging to other specialities, nor the utility of other bedside examinations and diagnostic predictors. We have aemended the text in the Discussion section to reiterate the aim of our work (Page 15, Line 247 - 248).

- **Comment 2:** *You compare HINTS against STANDING, but HINTS is about AVS but STANDING is about Differential Diagnosis of Vertigo in the Emergency Department, it includes for example, another etiologies of vertigo like BPPV so a comparison is not possible.*

**Response:** Both the STANDING algorithm and the HINTS examination are two diagnostic algorithms specifically developed for vertigo examination and the evaluation of dizziness.^[1]^ The Gerlier et al. study also explicity compares the HINTS examination with the STANDING algorithm.*^[2]^* We did not discuss etiology because this is beyond the scope of our work. We note that Tarnutzer et al. (2011) have published an extensive list of the differential diagnoses of acute vestibular syndrome, based on expert opinion.^iii^

Our only objective was to evaluate whether frontline point-of-care emergency physicians, without specialist equipment, can diagnose central vertigo in AVS using the HINTS examination or its variants with sufficient diagnostic accuracy and reliability. We have amended the Introduction section to emphasize that we did not set out to make any comparisons (Page 4, Line 64 - 65).

**Additional Clarifications:**

In addition to addressing the issue above, we made several other amendments to our submission, as follows.

1. We made moderate revisions to the manuscript in terms of formatting, language and grammar. We have also made some sentence reconstructions and word choice changes for accuracy and enhanced clarity. These revisions have not been tracked.
2. We updated the author’s affiliations (Page 1).
3. We corrected the year associated with ATTEST (to 2017) and selected another study as the reference (Page 3, Line 44 - 45).
4. We made minor revisions to Figure 1 and added captions (Page 7).
5. We made moderate revisions to Table 1 and added captions (Page 11, 12).
6. We made moderate revisos to Figure 2 and added captions (Page 12). We believe the information required was reflected well in our traffic plot (the summary plot was not required).
7. All figures have been modified by the Preflight Analysis and Conversion Engine (PACE) digital diagnostic tool.
8. The minimal underlying dataset has been submitted to The University of Sydney eScholarship Repositary. This has been reflected within the manuscript (Page 10, Line 181 -182). We will contact you as soon as our dataset is online to revise refence number 30 (Page 23).
9. We removed the supplementary material as it is now available in the data set.

1. Vanni S, Pecci R, Edlow JA, Nazerian P, Santimone R, Pepe G, Moretti M, Pavellini A, Caviglioli C, Casula C, Bigiarini S, Vannucchi P, Grifoni S. Differential Diagnosis of Vertigo in the Emergency Department: A Prospective Validation Study of the STANDING Algorithm. Front Neurol. 2017;7;8:590. doi: 10.3389/fneur.2017.00590. PMID: 29163350; PMCID: PMC5682038.
2. Clin Trials.gov US National Libraray of Medicine; 2019. Eye-ECG approach to emergencies: diagnostic performance of the HINTS test; [cited 2020 Jul] https://clinicaltrials.gov/ct2/show/NCT04118361 ID: NCT04118361
